# Algal Biomass Utilization toward Circular Economy

**DOI:** 10.3390/life12101480

**Published:** 2022-09-23

**Authors:** Magdalena Zabochnicka, Małgorzata Krzywonos, Zdzisława Romanowska-Duda, Szymon Szufa, Ahmad Darkalt, Muhammad Mubashar

**Affiliations:** 1 Faculty of Infrastructure and Environment, Czestochowa University of Technology, Dabrowskiego 69, 42-201 Czestochowa, Poland; 2 Process Management Department, Wrocław University of Economics and Business, Komandorska 118/120, 53-345 Wrocław, Poland; 3 Department of Plant Ecophysiology, Faculty of Biology and Environmental Protection, University of Lodz, Banacha 12/16, 92-237 Lodz, Poland; 4 Faculty of Process and Environmental Engineering, Lodz University of Technology, Wolczanska 213, 90-924 Lodz, Poland; 5 Department of Renewable Natural Resources and Ecology, Engineering Agricultural Faculty, Aleppo University, Aleppo 12212, Syria; 6 College of Life Sciences, University of Chinese Academy of Sciences, Beijing 100049, China

**Keywords:** circular economy, algae, biofuels, biomass, CO_2_ capture, wastewater

## Abstract

A review of the potential areas of algal biomass utilization has already been conducted. In addition to lowering the greenhouse effect and contributing to the decrease in the amounts of harmful substances in the air and water, attention has been paid to the possibility of utilizing algal biomass as a feedstock for the production of environmentally friendly products. The circular economy addresses the benefits to the environment, economy and society. The utilization of algal biomass benefits the environment by reducing greenhouse gases emissions as well as water and wastewater treatment, benefits the economy by producing biofuels, and benefits society by producing food, cosmetics, pharmaceuticals, fertilizers and feed for animals.

## 1. Introduction

The circular economy (CE) addresses the benefits to the environment, economy and society. The concept of CE is linked to the Sustainable Development Goals by reducing consumption and achieving savings of raw materials, water and energy. The utilization of algal biomass benefits the environment by reducing greenhouse gases emissions as well as water and wastewater treatment, benefits the economy by producing biofuels, and benefits society by producing food, cosmetics, pharmaceuticals, fertilizers and feed for animals.

Microalgae is a diverse group of unicellular organisms that are the ancestors of plants [1]. They can be seen as a potential solution to the problem of the demand for liquid fuels. Species of algae inhabit various environments from freshwater (about 40% of identified species) are from the freshwaters to saturated saline (more than 50% are from the marine water) [2].

Most microalgae are autotrophs, but they are also capable of producing energy in a heterotrophic or mixotrophic manner. One of the adopted divisions distinguishes the following groups of microalgae: *Bacillariophyceae*, *Chlorophyceae*, *Phaeophyceae*, *Myxoophyceae*, *Chrysophyceae*, *Rhodophyceae*, *Xanthoophyceae*, *Cryptophyceae*, *Dinophyceae*, *Euglenophyceae*, *Chloromonadinae* [3]. Recently, the interest in microalgae has increased significantly due to their very high rate of biomass growth and the possibility of using it in many industrial fields [4].

Microalgae are characterized by a very fast growth rate and can close their entire life cycle within a few days. In their breeding, the most important thing is to provide light energy and a source of biogenic elements, mainly nitrogen and phosphorus [5]. The cultivation of algae for energy purposes is characterized by high efficiency, which is much higher than the cultivated energy crops (20 times faster growth of algae biomass compared to the biomass obtained from maize or rape). In addition, algae cultivation can be carried out in bioreactors on soil fallow that do not meet the criteria in the valuation scale for plant crops [6,7].

One of their essential advantages is the effective use of CO_2_. Notably, they account for more than 40% of the global carbon fixation, and most of this productivity comes from marine microalgae [8]. Microalgae produce oxygen and contain chlorophyll *a*. They are mainly autotrophs that use atmospheric carbon dioxide (CO_2_) as the primary source of carbon. Some of them are mixotrophs, which use CO_2_ and also organic carbon. They may even grow as heterotrophs and use the previously fixed carbon as a carbon source [1]. Photoautotrophic algal growth depends on a light needed for photosynthesis, while heterotrophic depends on an organic carbon source [9].

Using two energy sources (organic carbon and light) leads to the flexible growth of microalgae in mixotrophy mode. This might help in achieving high growth rates and biomass productivity. Mixotrophy also reduces biomass loss during night-time because microalgae use organic carbon instead of stored carbohydrate for catabolism [10].

To date, there have been more than 100,000 identified strains of algae. They contain lipids (max. 40% on a weight basis), carbohydrates, and proteins (up to 50% of its dry weight) in different proportion [11]. Lipids can be separated and used as the primary feedstock for biodiesel production [12]; carbohydrates can be starch-fermented into ethanol. The remaining proteins might be added to animal fodder [13]. Algae might be used as food supplements and nutrients for human, livestock feed, fine organic chemicals for pharmaceuticals, pigments, and various other applications [11].

Many factors affect the production of a variety of high energy molecules as well as microalgae biomass. Among them, carbon and nitrogen sources used for cultivation, C/N ratio, the availability of other nutrients, such as phosphorous, and environmental conditions are the most critical [10]. Microalgae can be grown in wastewater that can be installed on land unsuitable for agriculture, and they have higher energy conversion efficiency than first- and second-generation fuels [14].

One of the elements determining algae-based biofuels’ economic viability and bioproducts is obtaining high-density biomass, the biochemical composition of cells, and the cultivation system [15]. In the case of algae, the bioproduct extraction of intracellular products is challenging. They cannot be effectively recovered using typical methods used, e.g., for soya, due to the different morphology.

The present review provides a comprehensive overview of various utilizations of algal biomass. The work scope included discussing the potential for reducing CO_2_ emissions due to the gas’s capture by algae. Algae use for wastewater treatment has been discussed, and the review of the algal biomass development has also been completed.

## 2. CO_2_ Capture by Algae

A significant source of CO_2_, a well-known greenhouse gas (GHG), is released into the atmosphere as an effect of fossil fuel combustion that is the primary source of energy in power [16]. The transportation sector accounts for 21% of the current global fossil fuel CO_2_ emissions to the atmosphere, second only to emissions from power production [13].

According to the US Energy Information Administration’s International Energy Outlook 2016, global energy-related CO_2_ emissions will increase from 32.3 × 10^9^ metric tons in 2012 to 43.2 × 10^9^ metric tons in 2040 [17]. A vast amount of CO_2_ is produced by the cement industry, which is accountable for about 8% of global CO_2_ emissions [10].

Although there are different CO_2_ capture approaches, the biological CO_2_ capture method is a potentially attractive alternative. The sustainable carbon circular economy is going to replace the “carbon to waste economy” [18]. Carbon dioxide can be converted by photosynthesis into organic matter by utilizing sunlight as a source of energy [16]. CO_2_ can be captured by algae from atmospheric, from power plants exhausted gases and industrial processes, and from carbonate [10]. Carbon assimilation in algae can be through the gaseous form of CO_2_ (diffusion through the algal cell membrane) or as dissolved bicarbonate (through bicarbonate transporters at pH 6.4–10.3) [10,19]. Using carbon dioxide from the atmosphere is likely to reduce the carbon footprint of algal fuels significantly. Unfortunately, no method exists for growing algae at high productivity using only the carbon dioxide available at the concentration in the normal atmosphere [20].

Some green algae, e.g., *Chlorella* species, are reported to be easily grown at very high CO_2_ concentrations. It is a very common alga to be used in carbon sequestration [10]. Concentrated carbon dioxide sources are mainly the flue gases produced during power generation from the combustion of coal. Because carbon dioxide contributes substantially (≈50%) to the cost of producing the biomass, algae culture for fuels is not feasible unless carbon dioxide is available free [20,21].

Microalgae is of high interest for CO_2_ sequestration, as the biomass can be used widely, such as supplementing as animal feed, biofertilizer or as a feedstock for biofuel; thus, it can introduce the resource recycling. There are several microalgal strains that have been tested in CO_2_ sequestration (Table 1). It can be seen from the table that the sequestration rate may range from 0.39 to 51.5 g L^−1^ d^−1^. This shows that the carbon sequestration potential of microalgal strains is directly affected by biomass growth and may also be depending on biomass composition depending on the strain. Furthermore, *Chlorella* sp. is the most preferred microalgal strain for CO_2_ sequestration studies due to its higher growth rate; Bhowmick et al. [22] reported a fivefold higher growth rate for *Chlorella minutissma* than *Euglina*. Overall, Table 1 further attests that microalgae could be an excellent agent for the forced CO_2_ sequestration, and the CO_2_ from waste flue gas of the industrial chimneys can be derived to the microalgal photobioreactors.

The use of algae to remove CO_2_ from power plant flue gases and yield valuable by-products as biodiesel has received significant commercial interest in Europe because of subsidization, profits from a greater volume of sales of biodiesel, and the application of residual algal biomass for further energy recovery [23]. The net CO_2_ benefit from algae is dependent on the emissions from the subsequent use of biomass as fuel [24].

Currently, the capture of CO_2_ by algae is much more expensive than CCS (Carbon Capture and Storage) technology, which consists of separating, capturing, and storing carbon dioxide from exhaust gases [20,25]. The average cost of producing 1 ton of dry algae biomass, which used CO_2_ from the power plant, is USD 500 for closed bioreactors and USD 110 for open ponds. The price of capturing one ton of CO_2_ by algae is USD 250 for photobioreactors and for open ponds USD 55 per ton of CO_2_ [20].

To sum up, the benefits to the environment by reducing greenhouse gases emissions are connected directly to the aims of the CE.

## 3. Wastewater and Water Treatment

The production of energy, recovery of nutrients, and fixation of inorganic carbon emitted in the atmosphere are possible by algae-based wastewater treatment [26]. Algae’s wastewater treatment needs to be technologically feasible, environmentally friendly, and economically viable [27]. Biological CO_2_ fixation using microalgae could be combined with other processes such as wastewater treatment. For instance, in exceptional situations, when discharging raw sewage or slurry into lakes, in a water tank, states unprecedented in nature, such as polytrophy and hypertrophy, are achieved [28].

**Table 1:** Microalgae for the fixation of CO_2_ during wastewater treatment.

Strain	Reactor Type	CO_2_ Source	CO_2_ Comp.	Growth Rate	CO_2_ Fix. Rate	Ref.
			%	g L^−1^ d^−1^	g L^−1^ d^−1^	
*Anabaena* sp.	Circular PBR	commercial	10	-	1.01	[29]
*S. dimorphus*	flat-panel *PBR*	comp. CO_2_	-	*-*	0.60	[30]
*Chlorella* sp.	Fabricated PBR	boiler gas	8	1.296	2.33	[31]
*C. minutissma*	cylindrical	-	5	0.293	51.51	[22]
*Coelastrella* sp.	Flask	commercial	1	0.80 ^b^	0.395	[32]
*C. sorokiniana*	flask	commercial	1	1.06 ^b^	0.567	[32]
*Scenedesmus*	flask	commercial	10	0.06 *^b^*	0.446	[33]

POME: palm oil mill effluent; ^b^: growth rate as maximum specific growth rate (μ_max_ (day^−1^)); Comp. CO_2_: compressed CO_2_.

However, the cultivation of algae does not harm the natural environment due to the lack of the need to use pesticides and other synthetic chemical compounds leading to disturbance of the ecosystem balance, including the extinction of species and the accumulation of xenobiotics in soil and water [34]. Algae are tolerant to a high concentration of carbon dioxide, which makes it possible to sequestrate it [4]. Increased CO_2_ concentration increases the rate of biomass growth and the lipid content in *C. vulgaris* cells [34,35,36,37]. In addition to carbon dioxide, algal growth requires nitrogen (N) and phosphorous (P) as principal nutrients [20]. Nitrogen compounds, especially ammonium (NH_4_^+^) and nitrate (NO^3-^), are essential substrates for microalgae. These compounds contribute to more than 10% of the microalgal biomass. Additionally, urea and nitrite are other forms of nitrogen compounds, but the latter is considered toxic at high concentrations [16].

Phosphorus is another crucial nutrient for microalgae growth. Phosphorus can participate in the formation of proteins, lipids, and intermediates of carbohydrates. Similarly, microalgae can incorporate inorganic phosphate compounds such as hydrogen phosphates (H_2_PO_4_^−^ and HPO_4_^2−^), forming organic species via phosphorylation. Furthermore, some microalgae can utilize phosphorus, forming organic esters, which are valuable for cell growth [16].

High levels of nutrients in the wastewater effluents cause eutrophication. During eutrophication, increased growth of algal biomass occurs. Therefore, instead of classifying algae as waste, they can be used for economic purposes. This would be advantageous to offer more economical feasibility and environmentally sustainable [16]. The use of algae in wastewater treatment processes, especially for removing biogenic compounds, i.e., nitrogen and phosphorus, is discussed in the literature [38,39]. For example, the microalgae *Chlorella vulgaris* grown on glucose-supplemented municipal wastewater reduced 96.9% chemical oxygen demand, 65.3% total nitrogen, and 71.2% total phosphate [9]. The usefulness of algae in the wastewater treatment processes is that nitrates and phosphorus compounds and carbon dioxide are used for algae growth and reproduction. The main by-product is oxygen. Algae absorb large amounts of biogenic compounds (nitrogen and phosphorus), as they are essential for the synthesis of protein constituting 20–60% of the algal mass. The absorbed biogenic substances are also necessary to form nucleic acids and phospholipids [40].

Wastewater treatment using algae offers significant advantages over conventional wastewater treatment. Oxygen is produced during microalgae photosynthesis, which provides disinfection and can significantly reduce mechanical aeration costs. This makes wastewater treatment more effective. In addition, the property of settling microalgae allows limiting the use of chemicals in the flocculation process. Algal biomass can also provide dual benefits by reducing nutrients and producing value-added products [9]. The technologies used in wastewater treatment by algae include adsorption, accumulation, and algae immobilization [26]. Specifically, municipal wastewater provides a good option for microalgae utilization. This is because municipal wastewater effluents are produced in large amounts and are rich in nutrients [16]. The use of algae for nutrient removal from municipal wastewater has been extensively investigated, and in general, this nutrient stream provides an excellent microalgal growth medium [1]. When algae are grown on industrial and agricultural wastewaters in high rate algal ponds, a low-cost by-product as algal biomass is obtained. The harvest costs should be included in the wastewater treatment operation [11,41]. The utilization of microalgae to remove the nutrients (mainly nitrogen and phosphorus compounds) from wastewater is a green technology that reduces or replaces the use of chemicals in wastewater treatment plants. Thus, this technology’s benefits allow both CO_2_ capturing through photosynthesis and removing nutrients from wastewater [16].

Ideally, when using microalgae-based systems for wastewater bioremediation, nutrients removal is needed, but their recovery is also targeted. In this way, nutrients are not lost but recycled in biomass that can be further valorized as fertilizer or substrate for bioenergy or bioproducts generation [42].

Microalgae growth and nutrients removal mechanisms involved in wastewater bioremediation are highly dependent on temperature and light. The optimal growth temperatures for most microalgal species range from 15 to 26 °C [36].

It is recommended to use the algal system rather than tertiary treatments, given its economic feasibility [43]. Optimizing algae growth in open ponds is crucial for reaching economic viability and remains a significant challenge for the industry [8]. Globally, more than 80% of algal biomass is generated in open ponds, which is mainly due to the low investment costs. However, the use of closed photobioreactors will grow by 2024 in terms of demand and sales [44].

Algae are also used in wastewater technologies for heavy metals’ biosorption as they are natural raw materials and are cheaper to produce than filter membranes or ionites [45]. It might occur as bioaccumulation, which uses the living cells’ accumulation abilities, or a biosorption process that occurs on non-metabolic cells [46]. Biosorption can be applied using different dry biomass types to remove metals in wastewater [47]. Microalgae cells have the potential to be utilized for heavy metal removal due to their ability to accumulate Hg, Cd, Zn, Au, Ag, Co, Mn, Cs, Ni, Fe, Cu, and Cr in their cells [44,48]. In addition, toxic metals such as Ni and Al can be removed from industrial wastewater by *Spirulina platensis* and *Chlorella vulgaris* strain. The improvement of the above-mentioned toxic metals’ removal efficiency by algae strains could be made by applying acidic treatment using sulfuric acid. The acidic treatment improves the algae strains’ surface properties by increasing the portion of the negatively charged functional groups on the algal biomass surface [49].

Lead, cadmium, copper, zinc, and chromium metals accumulated in algal cells can account for more than 25% of the algal dry matter. *Spirulina platens* has the highest capacity to remove cadmium from water, and other species of microalgae such as *Scenedesmus quadricauda*, *Pseudochlorococcum* are involved in the removal of mercury, cadmium, and lead [50]. Some strains (*Nannochloropsis* sp., *Chlorella vulgaris*, and others) tolerate heavy metal contamination up to a maximum value of 1000 ppm [23].

**Figure 1:**
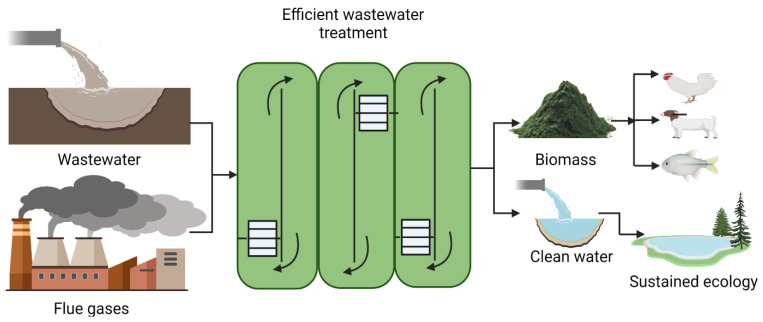
Environmental benefits from cultivation and utilization of algal biomass (created with BioRender).

Microalgae are considered efficient in wastewater treatment as microalgae use nitrogen and phosphorus from waste resources and convert them to biomass. Figure 1 shows a schematic view of the integration of microalgae with wastewater nutrient removal. Microalgae are capable of removing nutrients from municipal, industrial, and dairy wastewaters along with CO_2_ sequestration when it is incorporated with wastewater treatment. Wastewater grown microalgae utilization as feed, food, or fertilizer serves the purpose of the circular economy principles. Furthermore, the nutrient removal rate and removal efficiency depend on microalgal strain or culture conditions. Table 2 compares the removal rate and removal efficiency of different microalgal strains from wastewater from different resources. The removal rate of N and P can range between 2.5 to 170 mg L^−1^ d^−1^ and 0.09 to 58.1 mg L^−1^ d^−1^, respectively, with removal efficiencies of nearly 100%.

Furthermore, exploiting growing microalgae and bacteria cooperatively can further enhance the wastewater treatment efficiency. The mutualism in microalgae and bacteria helps each other, and thus, the overall efficiency of the system is higher than their individual counterparts. For example, microalgae provide bacteria the organic compounds and O_2_ that are released during photosynthetic microalgal growth. In return, bacteria provide microalgae with CO_2_ and organic elements that works as growth enhancers for microalgae. The mutualism has been experimentally proved; Perera et al. [50] exploited a consortia of *T. obliquus* and *V. paradoxus* for dairy wastewater treatment and reported N and P removal efficiencies of 78.61% and 87.6% for sole T. obliquus, whereas the removal efficiencies were 100% and 92.2% in case of T. obliquus and V. paradoxus consortia. This 22% and 4% increase in N and P removal efficiency was reported due to the production of neutral lipids that indicate significant mutualistic interaction. Although these show higher returns of microalgae-bacteria consortia for nutrient removal, the selection of microalgae-bacteria consortia requires the careful selection of microalgal and bacterial strains for their specific role in nutrient removal and restricting the density of competing strain accordingly.

The benefits to the environment and society by the utilization of algal biomass for water and wastewater treatment are linked to the circular economy.

**Table 2:** Summarizing nutrient removal from different wastewater using microalgae and microalgae-bacteria consortia.

Strain	Wastewater	Working	Light	Time	Growth Rate Removal Rate			RE		
	Type	Volume	μmol/m^2^/s	Days	g L^−1^ d^−1^	(mg L^−1^ d^−1^)		(%)		
		L				N	P	N	P	Reference
*N. aquatica*	swine	0.2	150	7	0.82	53 ^a^	58.1 ^a^	96.2	46.3	[51]
*Coelastrum* sp.	dairy	0.04	42.55 ^b^	10	0.266	2.55	2.31	84.7	100	[52]
*A. oryzae* and	starch	-	30	3	-	170.1 ^c^	15.7 ^c^	83.56	96.58	[53]
*C. pyrenoidosa*										
*C. sorokiniana*	acid prod.	0.5	NL	7	0.75	83.64	5.51	88.05	82.69	[54]
*C. pyrenoidosa*	dairy	1	-	8	0.08	13.25 ^c^	1.80 ^c^	97.31 ^d^	90.25	[55]
*Scenedesmus* sp.	Domestic	0.25	28	10	-	5.87 ^c^	0.091 ^c^	93.81	91.04	[56]
*C. vulgaris*	sewage	50	555–1850 ^b^	13	0.067	4.8	1.4	92.3	77.7	[57]
*T. obliquus*	dairy	0.25	-	8	-	5.48 ^c^	6.98 ^c^	78.61	87.61	[50]
*T. obliquus*	dairy	0.25	-	8	-	6.97 ^c^	7.35 ^c^	100	92.2	[50]
*V. paradoxus*										
*Chlorella* sp.	slurry	0.3	46.25 ^b^	10	113	17.80 ^c^	2.11 ^c^	82.07	79.6	[58]
*Lysinibacillus* sp.										

^a^: N as ammonia; ^b^: converted from lux to μmol/m^2^/s; ^c^: removal rate calculated from initial concentration and removal efficiency; ^d^: RE is sum of ammonia and nitrate. NL: No light source.

## 4. Liquid Biofuels

Algal biomass (third-generation biofuel sources) might be a solution for rising global demands for transport fuels. Algae offer great potentials as a biomass resource for green transport fuels and direct use in carbon sequestration [13,36]. That offers benefits to the CE.

Bioenergy deriving from the combustion or processing of algal biomass is becoming more popular [11]. The idea of producing biofuels from microalgae is not new, but it is gaining more and more people interested in combating global climate change [59].

The cultivation of algae does not harm the environment due to the lack of the need to use pesticides and other synthetic chemical compounds leading to the disturbance of the ecosystem balance, including the extinction of species and the accumulation of xenobiotics in soil and water [34]. Algae are tolerant to a high concentration of carbon dioxide, which makes it possible to sequestrate it [4]. Increased CO_2_ concentration increases the rate of biomass growth and the lipid content in *C. vulgaris* cells [34,36,37].

Renewable electricity generation systems are being deployed rapidly; however, renewable fuel technologies are ≈10–20 years behind on the development curve [60]. Based on the feedstock used for production and the technologies used to convert that feedstock into fuel, biofuel technologies could be classified into three groups: first-, second-, and third-generation biofuels [61].

The amount of oil obtained from the algae in the area of 1 hectare is significantly higher than that of the crop, and the critical area under cultivation is, in turn, significantly smaller [20].

The lipid content of microalgae varies considerably for different species. The compositions and fatty acid profile of lipids extracted from a particular species are affected by the cultivation conditions, such as medium composition, temperature, illumination intensity, the ratio of light/dark cycle, and aeration rate and ranges from 12 to 22 carbons in length [62].

Algae with the highest oil content include the species of *Botryococcus braunii* and *Schizochytrium* sp. The oil content of these species reaches up to 70%. The species with the lowest oil content (up to 30%) are *Crypthecodinium cohnii*, *Dunaliella primolecta*, *Nannochloris,* and *Tetraselmis sueica* (Table 3). *Pseudokirchneriella subcapitata* is a high-yield source of fatty acids and a potential oil source for biodiesel [63].

Algal oil can be successfully produced as an algal–biomass chain co-product from biological capture systems in the industrial power plants and later be used as a fuel for engines. Algae oil is a triglyceride, and it can be further converted into biofuels such as biodiesel through the same processes used to convert plant oils [23]. Nowadays, Algenol (Fort Myers, FL, USA) and PowerFuel.de (München, Germany) are producing algae-based oil. However, new technologies and ideas are still required to increase more oil production, replacing existing fuels [64].

The difficulties in efficient biodiesel production from algae are caused by not using an algal strain with a high lipid content and fast growth rate, problems with harvesting, and a cost-effective cultivation system needed for concentrated CO_2_ [13].

Several microalgal strains accumulate carbohydrates mainly as insoluble starch and cellulose. Microalgal biomass is not readily accessible to common fermenting microorganisms (e.g., bioethanol synthesis). However, it would be potentially easier to convert it into monosaccharides than plant lignocellulosic materials because of the lack of lignin [66].

Regarding downstream processing, microalgal carbohydrates content (quantity and quality) and pretreatment are among the most critical variables toward the competitive production of bioethanol. It is estimated that pretreatment would account for as much as one-third of the total cost of algae bioethanol production. In addition, for economically-competitive ethanol production, a minimum of 40 g ethanol per liter of fermentation broth would be needed to reduce distillation costs [66].

About 98% of commercial algae biomass production is carried out in open ponds. The obtained high-value nutritional products are sold for over a hundred and even a thousand-fold higher than allowable for biofuel [13].

Approximately 60–75% of the total cost of microalgal biodiesel comes from microalgae cultivation, which is mainly due to the high cost of the carbon source, the fertilizer requirements, and the high cultivation facility costs relative to often low oil productivity [67]. The net revenues from a 100 t per ha year microalgae production system range from 210 to 415 EUR [13]. In addition to their ecological and economic benefits to the transport and energy sectors, oils cannot be used directly in diesel engines due to their too-high viscosity for modern high-pressure pumps [23].

The development of the first and second-generation biofuels has benefited mainly from various policy interventions, e.g., directly supportive measures (tax concessions, reduced fuel excises, and subsidies for production and infrastructure); or indirect measures (biofuel blending mandates and trade measures protecting domestic biofuel industries from lower-cost foreign suppliers) [24]. Transitioning for third-generation biofuels managing the associated risks is considered a significant challenge regarding the costs and technological developments required [24].

Microalgal biomass is a potential source of energy and bioproducts because of its ability to produce approximately 300 times more renewable oil [64]. Liquid, solid and gaseous biofuels from algae may become commercially available in the years 2020–2025 [23].

Algae as the fuel source have some negative factors that impact the high price, small payback, and low popularity. Expensive production and extraction processes determine the high price of an end-product, while production payback depends on algae species, growing method, conditions, extraction method, and others [23].

Algal biofuel production from wastewater treatment can provide the sustainable environmental benefits of sequestering CO_2_ and significantly impact the environment in terms of water footprint, energy and fertilizer use, and residual nutrient removal compared to commercial algal production, which consumes freshwater and fertilizers. In addition, it offers significantly better economics for plant capital and operation costs [9,11].

After biofuels production, organic N and P in algal wastes can be mineralized to a flux of ammonium and phosphate, either recycled as a substrate for microalgae growth or sold soil conditioners and fertilizers. The water used during the cultivation of algae can be recycled into algae growing systems. Thus, from a sustainability viewpoint, the processing water and nutrients (N and P) can be recycled, and the recovery of bioethanol and biogas can potentially result in an energetic balance of the microalgae to biofuels process, which can improve the economics of the algal biorefinery approach [59].

## 5. Gaseous Biofuels

The production of gaseous biofuels from algal biomass helps to implement the circular economy aims, too. Methane is a very good energy carrier. As a result of burning 1 m^3^ of this gas, 39.7 MJ of energy is generated, which means that it can be effectively used as fuel in transport. In Europe, the most biogas is produced in biogas plants during methane fermentation of maize biomass (*Zea mays*), although high hopes are also associated with hemp (*Cannabis sativa*). The law in some countries, including Poland, significantly limits the use of this plant in cultivation. An alternative may be to produce algae biomass from the waste and then digest it with methane fermentation. Wastewater treatment ponds are currently the most economic approach to the production of microalgal biofuel. Heterotrophic culture may be preferred over photoautotrophic cultivation. Contrary to the production of biodiesel, the process of drying and extraction can be omitted in the production of methane, which significantly reduces costs, and it consequently uses all the compounds present in the cell: sugars, fats, nucleic acids and proteins, which is not the case for biodiesel. The high protein content of the algae cells can result in the formation of ammonia, which is toxic in high concentrations. In order to reduce the concentration of this metabolite and at the same time increase the intensity of biogas production, algae cultivation can be combined with the disposal of other industrial waste, which is rich in carbon compounds but poor in nitrogen compounds: for example, by adding waste paper, mainly composed of cellulose [68,69,70]. Unfortunately, the production of methane on an industrial scale using algae is still not profitable. The process should be optimized by, for example, the construction of more efficient systems for the cultivation of algae, the selection of the appropriate bacterial strain for fermentation and the algae species itself, which is tolerant to stress caused by an unfavorable environment, as well as the integration of technology, i.e., placing the culture near industrial plants from which it is possible to extract heat or carbon dioxide [36,68,71].

Hydrogen is a desirable substrate in the chemical industry, as it is needed for many processes, and it can react with many toxic pollutants and decompose them into non-toxic forms. It is also a good source of energy, and the heat of its combustion from 1 m^3^ gives 12.6 MJ and can be used as a biofuel. Hydrogen is produced during the synthesis reaction, thermochemical reactions, and in the metabolism of living organisms. The last method of producing hydrogen is the least harmful to the environment as it does not produce toxic chemical waste. When total hydrogen is burned, no carbon dioxide is produced, but only water and heat are released. Biohydrogen can be obtained by fermentation using bacteria, but cyanobacteria and algae can also be used [72,73]. Microalgae can produce hydrogen by the direct photolysis of water if grown under light and anaerobic conditions using the enzyme hydrogenase. Cyanobacteria can also produce hydrogen by the indirect photolysis of water using the same enzyme. The amount of hydrogen obtained is greater as a result of the second process, but it is also more difficult to carry out because continuous lighting and the addition of ATP are necessary [69].

## 6. Food

Since the CE aims at the prevention of environmental degradation while ensuring the economic and social well-being of the present and future generations, then the food sector is also looking forward to a CE [74]. The implementation of a circular economy can reduce resource consumption and emissions to the environment by moving away from a linear and unsustainable system [75,76]. Algae are widely used in the food industry that bring benefits to the society, which is one of the aim of the CE. The high protein content of most microalgae and their amino acid composition make them suitable for human and animal nutrition [8]. Hydrocolloids (biopolymers) obtained from algae give the product to which they have added a new, more stable structure [77]. Phycocolloids (carrageenan, agar, and alginates) can be used as emulsifiers, viscosifier, and gelling agents and are attractive for science and industry [78].

Brown algae produce alginic acid (E400) and sodium alginate (E401) have been used in the manufacture of ice cream, desserts, yogurts, puddings, vegetable, and fish canned concentrates, drinks and cakes, mayonnaise, broths and soups, products with reduced sodium contents, and beer [5].

Carrageenan (E407), a naturally occurring substance in algae, has been used to produce cake glaze, sugar-free desserts, jellies, sorbets, and jellies with fruit, or mayonnaise imitation. As a thickener, carrageenan is used to produce fruit beverages [79]. The carrageenan’s viscous property makes it more valuable in the dairy industry, meat processing, and other miscellaneous products such as toothpaste, air freshener gels, and pet food [78].

Currently, there are a lot of dietary supplements and functional foods based on algae and cyanobacteria on the market. These products are characterized by high nutritional value and are a source of vitamins (mainly from groups B and A), proteins, antioxidants, minerals (including iodine), fiber and fatty acids—EPA (eicosapentaenoic acid), DHA (docosahexaenoic acid), and HUFA (highly unsaturated fatty acids). They are available in the form of powders, capsules or tablets [20,63]. A wide range of algae-based food products is particularly popular among bodybuilders who, due to their specific diet, have a high protein requirement [80,81].

The interesting aspect is the cultivation of heterotrophic in bioreactors with support by a primary carbon feedstock recovered from food waste. It could help to reduce environmental impacts and support the transition toward circular food systems [82].

The consumption of algae also raises some controversy if the cultivation of algae is not well controlled. In the event of improper cultivation, toxins, heavy metals, and secondary metabolites that may cause allergies may accumulate in the cells of the algae. In general, however, the consumption of algae in moderation, grown in appropriate conditions, is considered safe [83].

## 7. Pharmaceuticals and Cosmetics

The benefits to the society that need cosmetics and pharmaceuticals produced from natural sources are possible to realize by the utilization of algal biomass. Many algae species, including *Nannochloropsis*, *Nitzschia*, *Laminaria*, *Macrocystis pyrifera*, *Ecklonia*, *Lessonia*, *Durvillaea*, *Chlorella*, *Dunaliella salina*, *Dunaliella bardwil*, *Haematococcus pluvialis*, *Ulva*, *Sargassum*, etc. have been used in the pharmaceutical and cosmetic industries [84].

Algae in cosmetics are valued thanks to the content of acidic polysaccharides such as alginic acid, alginates, laminarin, carrageenan, fucoidan, and mannitol. In contrast, in the pharmaceutical field, they are valued for their antibiotic, anti-inflammatory, and anti-cancer properties [84].

Marine algae (mostly brown and red algae) contain cytostatic components with anti-cancer activity. Glycoproteins present in algae are used to reduce cholesterol and blood pressure. They also have anti-inflammatory and anti-depressant properties [84].

A wide variety of nutrients and secondary metabolites produced by microalgae are beneficial for humans or animals. Valuable current or potential co-products include carotenoids (such as lutein, zeaxanthin, lycopene, bixin, β-carotene, and astaxanthin) and long-chain polyunsaturated fatty acids [8].

In 2013, the world production of algae for consumption was estimated at $6.7 billion, and the main producers were India and China (Food and Agriculture Organization of the United Nations, 2015). *C. vulgaris* is a delicacy in Japanese cuisine and has a proven pro-health effect, i.e., stimulating the immune system, anti-cancer properties, reducing the chances of developing cardiovascular diseases and cataracts, and lowering blood pressure [85].

*Chlorella* sp., a rich source of chlorophyll, is especially suitable for people with liver problems or for smokers and is very helpful for people with bowel problems. It is also recommended for the people who need to regenerate and those suffering from degenerative diseases (muscular diseases, nervous system diseases). *Chlorella* sp. strengthens the immune system, digestion, helps to detoxify the body, accelerates recovery, protects against radiation, relieves pain during arthrosis [86].

There is actually a strong market demand for selected microalgal high-value products, including carotenoids, fatty acids, and phycobiliproteins. Currently, many producers such as Blue Biotech (Germany), Soliance (France), and BioReal (Sweden) are supplying microalgae products in the form of dietary supplements (*Chlorella, Spirulina*, and astaxanthin), cosmetic products, anti-inflammatory products, and slimming products in the market. Thus, the continuous bloom of microalgae in the future may fulfill the demand for essential components of food, feed, energy, pharmaceuticals, and cosmetics [64].

Microalgal production costs are still high; thus, it is hard to meet requirements for larger volumes at lower prices. In addition, the alternative sources for these products are available at lower costs, which limits the potential of microalgae products to niches such as vegetarian EPA (eicosapentaenoic acid) and DHA (docosahexaenoic acid) and natural carotenoids (vs. synthetics). Technological innovation is essential for microalgae transformation in process improvement and lower-cost production [44].

## 8. Animal Breeding

The livestock feed is another useful product that may be obtained from the algae. Comprehensive nutritional and toxicological evaluations have demonstrated algae biomass’s suitability as a valuable feed supplement or a substitute for conventional animal feed sources [11,87].

More and more often, attention is paid to the balanced composition of animal feed because of the implementation of the CE politics. Due to the high nutrient loss in animal feed, mineral salts (nitrates, chlorides, and sulfates) are used as feed additives. Unfortunately, their bioavailability is low. Therefore, it appears that a more effective form of animal micronutrient delivery is a bound biological matrix applied in the process of biosorption [46].

Algal cells are small with a relatively thick cell wall [88]. In the biosorption process, the passive attachment of micronutrients into the algal cell wall results in the increased micronutrient assimilability. Thanks to algae’s biosorption characteristics, it is natural to improve the feed’s composition with the necessary macro- and micronutrients. *Enteromorpha* sp. and *Chlorella* sp. are the most commonly used algae in animal nutrition [46]. More than 50% of the world’s *Spirulina* production is used as an animal feed supplement [89]. Astaxanthin is a red pigment that is mainly used as a feed additive for coloring salmon, carp, red sea bream, shrimp, and chickens.

In order to increase the absorption of nutrients from microalgae, processes that disrupt the integrity of the cell wall and facilitate the activity of digestive enzymes can be used [90]. It has been proven that the protein derived from *Spirulina* sp. after drying in the sun is absorbed by salmon in more than 80%, which means that it is a good source of protein that can compete with other fish food [91]. The dried algae feed (*Nannochloropsis* or *Tisochrysis lutea* species) compared to the fish meal and soybean feed provided the same growth rate for the fish in the Nile Tilapia culture. Moreover, meat from Nile Tilapia grown on food from algae was characterized by a composition of fatty acids that was more beneficial to the human diet [92,93]. The use of *Schizochytrium* algae as an addition to the diet of salmon (11% of total feed) did not change the levels of fatty acids (DHA) in the meat, but it reduced the amount of organic pollutants (including polychlorinated biphenyls). Moreover, it did not affect the growth rate of salmon or the organoleptic characteristics of its meat in any way [94,95]. The high content of colored chemicals in algae cells makes them used to create food dyes and dyes. When used as food, these compounds (chlorophylls, carotenoids) accumulate inside animals, so you can buy, for example, fish with orange scales [96]. Microalgae can be used as feed and provide stability in the food market in the future [97,98].

Nowadays, more than 70 companies were involved in the cultivation of *Chlorella*. The largest producer is Taiwan Chlorella Manufacturing and Co. (Taipei, Taiwan), which produces 400 tons of dried biomass per year [89].

## 9. Fertilizers

Initially, algae were used to fertilize soils near their place of occurrence, providing benefits to the environment, society and economy. Later, they were used to obtain relevant extracts, which contributed to the widespread use of this fertilizer type. Research on algal extracts indicates their beneficial effect on the cultivation of fruit, vegetables, and other plants. Their application has shown improved yields as the extract increases the plants’ resistance to adverse weather conditions (frosts or soil degradation), improved resistance to pathogens and pests, and increased nutrient uptake from the soil [99].

The recovery of N and P fertilizers in the effluent of anaerobic digester can improve the energy ratio of algal crude oil [20]. At a relatively high pH medium, phosphates sediment in phosphoric salts and organic matter [16].

Algae that have beneficial effects on the development of plants most often include green algae (Chlorophyta): *Cladophora dalmatica*, *Enteromorpha intestinalis*, *Ulva lactuca*, red algae (Rhodophyta): *Corralina mediterranea*, *Jania rubens*, and *Pterocladia pinnata*, as well as brown algae (Phaeophyta): *Ascophyllum nodosum*, *Ecklonia maxima*, *Sargassum* spp. [100].

Extracts from the algae mentioned above have properties that improve crops due to the presence of plant hormones (auxins, cytokines, etc.) in their composition, with the most significant influence of cytokines [101].

Algae can be used as fertilizers and plant biostimulants because they are a source of macro- and microelements, sugars, amino acids, and plant hormones (including cytokinins and auxins) [102,103]. They induce the formation of substances important for plants necessary for growth and allelopathic compounds, increasing yield and plant resistance to stress [87]. Moreover, the use of biopreparations, unlike their synthetic counterparts, does not harm the environment, and renewable energy sources are used for their production [104]. It has been shown that the use of *C. vulgaris* biomass as a fertilizer improves the soil structure and improves the germination capacity and speed of corn and wheat seeds [105]. Currently, preparations based on algae are available on the market, whose task is to support the growth and development of plants, for example: Maxicrop [106], Bio-Algeen, Kelpak [107].

The benefits of seaweeds application in the field are numerous, e.g., stimulation of seed germination, enhancement of health and growth of plants, namely shoot and root elongation, improved water and nutrient uptake, frost and saline resistance, biocontrol and resistance toward phytopathogenic organisms, remediation of pollutants of contaminated soil and fertilization [108].

The most commonly used in agriculture is Kelpak, which is an *Ecklonia maxima* algae extract from the South African coast. This extract’s chemical composition depends on the time of the year in which the algae were harvested. In the spring season, the product contains more cytokines and nutrients, while in the fall season, there is an increased number of antifungal agents and polyphenols. It stimulates plant growth and is widely used in vine and citrus farming, agricultural crops, and ornamental plants [101]. Bio-algeen S90 Plus 2 is marine algae extract, which improves plants’ rooting and increases resistance to stress and resistance to pathogen attacks [109,110].

## 10. Pros and Cos of Algae Production

Reducing consumption and achieving savings of raw materials, water and energy is possible by the utilization of algal biomass that is linked to the concept of CE as well as to the Sustainable Development Goals.

Apart from being a potential feedstock for biofuel production, algae play an important role in environmental pollution control, human health, animal and aqua nutrition, the cosmetics industry, the pharmaceutical field, and as a source for bioactive compounds, biomedical components, and high-value pigments [111]. Algae grow 20–30 times faster than food crops, contain up to 30 times more fuel than equivalent amounts of other biofuel sources such as soybean, canola, jatropha, or even palm oil, and can be grown almost anywhere [13].

The average total production cost for crude bio-oil is from USD 76.98 per gallon to USD 109.12 per gallon for the photobioreactor and for the open race pond system, respectively [15]. Reducing costs is crucial for commercial success. An outstanding, significant economic challenge for algal producers is identifying low-cost oil extraction and harvesting algae methods [11]. An efficient biomass fractionation and utilization is of absolute importance for lowering the production costs of the algae biomass. The market opportunity of any co-product intended must be carefully considered as must the life-cycle environmental impact of the strategy [44].

From the energy point of view, when algae biomass is cultivated, the most attractive case would be to utilize the waste heat left with the flue gas [23]. Despite the workable net energy and cost-effectiveness of multiple co-product and by-product approaches, microalgal biofuels are still not being produced at any scale and are dependent on subsidies [24,112]. The development of microalgae biofuel industries presents many socio-economic benefits that may contribute to a socially sustainable outcome, such as the generation of employment and economic growth in rural communities and an opportunity for economic growth in non-metropolitan and regional areas [24].

Microalgae have a high growth rate and very high yield per acre, lower demand for water than commercial crops, and high efficiency in CO_2_ mitigation. Algae are very energy and oil-dense; they are non-toxic, do not contain sulfur, and are very biodegradable [13,65]. Algal biomass technologies can contribute to social sustainability through employment and income generation, particularly for regional communities that are typically dependent on seasonal industries [24].

One of the critical bottlenecks for the production of biodiesel from microalgae is the small size of cells (less than 10 μm in diameter), their low concentration in the culture medium, and additionally negatively charged surfaces of microalgae, which prevents the easy deposition of these organisms by gravity [113,114].

A drawback of microalgae cultivation and processing is that they are capital and resource-intensive. Aside from the construction and maintenance of artificial environments, there are essential requirements for energy, water, and related nutrients for the facility to produce sufficient biomass [24]. The first challenge is separating water from the biomass due to small algal cells of 2–10 μm in length and 2–8 μm in width [100]. The difficulty of developing simple and inexpensive procedures to convert lipids into biodiesel [65,115], which can represent 20–57% of the final biomass cost, is also a bottleneck [1,8]. The cultivation of microalgae is not efficient from the energy point of view and needs more synergies.

## 11. Conclusions

Due to their particular chemical composition and properties, algae have gained increasing interest both from the scientific community and various industries. Remarkably, the following specific conclusions can be drawn:

1. Algae contain many micro- and macroelements that can be used in various areas of life.
2. Algal biomass could be utilized toward the circular economy and bring benefits to the environment, economy and society.
3. Their breeding process allows for the reduction in CO_2_ pollution by the binding of this gas from exhaust gases through algae cells in photosynthesis.
4. The culture can be carried out using wastewater purified by algae from biogenic compounds, heavy metals, etc.
5. It may be applied, for instance, in modern eco-construction, where algae can be used for household wastewater treatment, and the biomass will definitely be applied, e.g., as biofuel for buildings’ heating.
6. Algae can produce biofuels and can also be utilized in biogas plants and the production of biohydrogen.
7. They can be used as food, cosmetics, pharmaceuticals and also as feed for farm animals and fertilizers.
8. The increasing interest in using algal biomass for further new applications toward the circular economy may be forecasted in the coming years.

## Figures and Tables

**Table 3:** Oil content in selected species of microalgae.

Microalgae	Oil Content (% d.m.)
*Botryococcus braunii*	25–75 (Chisti 2007)
*Chlorella* sp.	2–32 (Chisti 2007) [64,65]
*Crypthecodinium cohnii*	20 (Chisti 2007)
*Cylindrotheca* sp.	16–37 (Chisti 2007)
*Dunaliella* sp.	6–42 (Chisti 2007) [64]
*Isochrysis* sp.	7–33 (Chisti 2007) [64]
*Monallanthus salina*	>20 (Chisti 2007)
*Nannochloris* sp.	20–35 (Chisti 2007)
*Neochloris oleoabundans*	35–54 (Chisti 2007)
*Nitzschia* sp.	45–47 (Chisti 2007)
*Phaeodactylum tricornutum*	20–30 (Chisti 2007)
*Schizochytrium* sp.	50–77 (Chisti 2007)
*Scenedesmus* sp.	1.9–40 [64,65]
*Spirulina* sp.	2–9 [64,65]
*Tetraselmis suecica*	15–23 (Chisti 2007)

## Data Availability

Not applicable.
